# Phasic Alertness is Unaffected by the Attentional Set for Orienting

**DOI:** 10.5334/joc.242

**Published:** 2022-10-07

**Authors:** Niklas Dietze, Christian H. Poth

**Affiliations:** 1 Neuro-Cognitive Psychology and Center for Cognitive Interaction Technology, Bielefeld University, Bielefeld, Germany

**Keywords:** phasic alerting, spatial orienting, attentional set, stimulus informativeness

## Abstract

Warning stimuli preceding target stimuli for behaviour improve behavioural performance, which is referred to as phasic alerting. Similar benefits occur due to preceding orienting cues that draw spatial attention to the targets. It has long been assumed that alerting and orienting effects arise from separate attention systems, but recent views call this into question. As it stands, it remains unclear if the two systems are interdependent, or if they function independently. Here, we investigated whether the current attentional set for orienting modulates the effectiveness of alerting. In three experiments, participants classified visual stimuli in a speeded fashion. These target stimuli were preceded by orienting cues that could predict the target’s location, by alerting cues that were neutral regarding the target’s location, or by no cues. Alerting cues and orienting cues consisted of the same visual stimuli, linking alerting cues with the attentional set for orienting. The attentional set for orienting was manipulated in blocks, in which orienting cues were either informative or uninformative about the target’s location. Results showed that while alerting generally enhanced performance, alerting was unaffected by the informativeness of the orienting cues. These findings show that alerting does not depend on the attentional set that controls orienting based on the informational value of orienting cues. As such, the findings provide a simple dissociation of mechanisms underlying phasic alertness and spatial attentional orienting.

## Introduction

Warning stimuli appearing shortly before visual targets improve behavioural performance by reducing reaction times (Callejas et al., 2005; Fan et al., 2002; Fuentes & Campoy, 2008; Hackley, 2009; Hackley & Valle-Inclán, 1998; Lin & Lu, 2016; Posner & Boies, 1971; Poth, 2020; at the expense of accuracy: McCormick et al., 2019; Posner et al., 1973) and/or increasing perceptual and response accuracy (Haupt et al., 2018; Kusnir et al., 2011; Matthias et al., 2010; Petersen et al., 2017; Wiegand et al., 2017). In this way, warning stimuli provide fundamental enhancements of human behaviour in time sensitive situations. The benefits provided by warning stimuli for perception and action are collectively referred to as alerting effects (Posner & Petersen, 1990). These effects are assumed to stem from a short-term increase in phasic alertness, the brain’s readiness for perceiving and responding to external stimulation (Posner & Petersen, 1990). Thus, phasic alertness reflects an intensity aspect of attention that refers to the momentary overall state of the attention systems (as discussed by Bundesen et al., 2015; Sturm & Willmes, 2001).

While attentional intensity affects stimulus processing in general (Bundesen et al., 2015), selective attention differentially affects the processing of task-relevant as opposed to task-irrelevant stimuli (Bundesen, 1990; Bundesen et al., 2005; Desimone & Duncan, 1995; Duncan & Humphreys, 1989; Eriksen & Eriksen, 1974; Lavie, 1995; Moran & Desimone, 1985; Treisman & Gelade, 1980; Wolfe, 1994). For instance, attention can be directed at specific locations in space, prioritising stimuli at these locations for perception and action (Carrasco, 2011; Eriksen & Hoffman, 1973; Hoffman, 1975; Posner et al., 1980; Yeshurun & Carrasco, 1998). This becomes evident in the spatial orienting effect, in which perceptual performance and reaction times are improved when orienting cues indicate where visual target stimuli are going to appear (Jonides, 1981; Posner et al., 1980; Posner & Cohen, 1984; Theeuwes, 1991).

Alerting and orienting have been assumed to rely on different processes implemented by separate brain networks (i.e., attentional networks) (Callejas et al., 2005; Fan et al., 2002, 2005; Petersen & Posner, 2012; Posner & Petersen, 1990). At the behavioural level, the evidence for or against a separation of alerting and orienting is mixed, sometimes suggesting additive effects (Botta et al., 2014; Chica et al., 2011) and sometimes suggesting interactions (Asanowicz & Panek, 2020; Callejas et al., 2005; Festa-Martino et al., 2004; Fuentes & Campoy, 2008; Karpouzian-Rogers et al., 2020; Lin & Lu, 2016). Although an interaction effect seems to persist across studies, its effect seems comparatively small (Chandrakumar et al., 2019). In sum, the relationship between alerting and orienting still remains unclear. A key question here is whether alerting is affected by the attentional set for orienting which defines the attentional priorities for visual processing.

Spatial attentional priorities seem influenced by the informativeness of a cue, which reveals that informativeness is an important source of information for the attentional set (Folk et al., 1992; Gibson & Kelsey, 1998; Vossel et al., 2006). In spatial orienting experiments, the stimulus informativeness is determined by the validity of orienting cues, that is, the probability that a target will appear at the cued location (Posner et al., 1980). As such, informative orienting cues (e.g., 80% validity) provide larger improvements of reaction times compared with conditions where the cues predict behavioural targets with uncertainty (e.g., 50% validity) (Eriksen & Yeh, 1985; Johnson & Yantis, 1995; Riggio & Kirsner, 1997). When the cues are always predictive of behavioural targets (e.g., 100% validity), they impose even stronger effects, because distracting involuntary shifts of attention seem not to occur (Folk et al., 1992; Theeuwes, 1991). Counter-predictive cues (e.g., 0% validity) require an extra controlled step of voluntary control as participants have to suppress the automatic attentional capture by the cue before they direct their attention towards the target location (Eckstein et al., 2004) which might diminish the impact of the attentional set.

Spatial orienting is controlled by the attentional set, which represents stimuli that indicate or predict where targets are going to appear (Awh et al., 2003; Leber & Egeth, 2006; Mulckhuyse & Theeuwes, 2010). Based on this information, mechanisms for orienting spatial attention can prioritise a given location at the expense of other locations (Bundesen, 1990; Bundesen et al., 2005; Desimone & Duncan, 1995; Duncan & Humphreys, 1989; Eriksen & Eriksen, 1974; Lavie, 1995; Moran & Desimone, 1985; Treisman & Gelade, 1980; Wolfe, 1994). The attentional set controls orienting by using spatial information but also by using information about non-spatial stimulus features, such as surface features (e.g., colour and shape; Wolfe & Horowitz, 2004, 2017) or luminance contrasts (Poth et al., 2014). Thus, surface features of stimuli that predict the locations of upcoming targets must be represented in the attentional set. The implications of such a representation of features in the attentional set are still largely unknown. For instance, the features in the attentional set could result in an enhanced processing of all external stimuli containing the feature. In this case, not only spatial orienting would be affected by the features in the attentional set, but other processes operating on stimuli containing the feature would be affected as well. As a result, the processing of alerting cues sharing surface features with orienting cues in the attentional set would be affected, either facilitating alerting effects through an improved processing or possibly impairing them through interference between the control of orienting and alerting. Rather than being isolated from alerting (Callejas et al., 2005; Fan et al., 2002; Fernandez-Duque & Posner, 1997), the top-down control of orienting would influence how external stimuli could regulate phasic alertness (and arousal). In contrast, however, if alerting and orienting were entirely independent, the attentional set for orienting should not affect the effectiveness of stimuli to act as alerts.

A first hint at an effect of the attentional set for orienting on alerting comes from a study by Lin and Lu (2016). They used a simple detection task whose targets appeared at one of two locations and could be preceded by stimuli at all possible target locations (double cues; i.e., alerting cues) or by a single stimulus at one of the possible target locations (single cues; i.e., orienting cues). These authors manipulated the spatial informativeness of the single cues in a between-subjects design, so that for one group of participants (the “informative group”), single cues validly predicted the upcoming target location on 80% of the trials, whereas for the other group of participants (the “uninformative group”), single cues were valid only on 50% of the trials. Single and double cues consisted of the same stimuli (black circles). It was supposed that the context-based task relevance of the stimuli depending on the informativeness of the single cues transferred to the processing of the double cue. Ultimately, this should have increased the alerting effects in the informative group compared with the uninformative group, which is exactly what Lin and Lu observed. Thus, alerting by the double cues could have been improved because double cues shared their surface features with single cues since the same stimuli were used for both. We should note, however, that alerting by double cues could have been improved also because double and single cues appeared at the same spatial locations. At the time of the cues, the informative group could have paid more attention to both potential locations (relative to the rest of the visual field), because the valid and thus task-relevant single cue was more likely to appear. In contrast, for the uninformative group, the single cue did not predict the target location, meaning that it was task-irrelevant so that participants had no reason to increase attention to the two possible cue locations. Thus, the informative group could have had larger alerting effects, because their alerting cues themselves received more attention compared with the ones of the uninformative group. Therefore, the findings cannot answer the question if phasic alertness depends on non-spatial features controlling the attentional set for orienting.

Here, we investigated the interplay between the attentional set for orienting and its effectiveness on alerting. Participants performed a speeded visual classification task whose targets were preceded by double cues, single cues or no cues. In Experiment 1, the attentional set was manipulated between blocks, in which single cues were either predictive (100% valid) or counter-predictive (0% valid) about the target’s location. The double cues and no cues were randomly intermixed within the two predictiveness blocks. Importantly, the double cues were presented at different visual axes than the single cues, so that both cue types were spatially separated. In Experiment 2, the single cues were either informative (80% valid) or uninformative (50% valid) about the target’s location otherwise it was identical to Experiment 1. In Experiment 3, the single cues were also informative (80% valid) or uninformative (50% valid), but the double cues were presented on the same visual axis as the single cues. If phasic alertness was influenced by the attentional set for orienting, both the alerting effect and the orienting effects should be greater in blocks with predictive than counter-predictive cues and greater in blocks with informative than with uninformative cues, respectively. In contrast, if alerting was independent from the attentional set for orienting, the differences between the predictiveness and informativeness blocks should only be found with single cues.

## Method

Experiments were conducted via the online platform Pavlovia (Open Science Tools Ltd., 2019) using the JavaScript framework jsPSych by de Leeuw (2015) with the PsychoPy application (Peirce et al., 2019). These tools have been found to offer a sufficient temporal precision for psychological reaction time experiments (Bridges et al., 2020). Participants performed the experiments online using their own computers, except for Experiment 3, whose participants performed the experiment in a seminar room of the university. They were instructed to set their monitors to a refresh rate of 60 Hz and use an external computer mouse for response collection.

### Participants

In Experiment 1, 41 participants aged between 19 and 42 years old (*median* = 24 years), 10 males, 1 diverse, and 30 females participated in the experiment in exchange for course credits. In Experiment 2, 42 participants aged between 16 and 60 years old (*median* = 25.5 years), 11 males, and 31 females received either course credits or had the chance to win a shopping voucher for participation. In Experiment 3, 71 participants aged between 18 and 35 years (median = 23 years), 16 males, 1 diverse, and 54 females received either course credits or were paid a compensation of 5 € for participation. A total of 12 additional participants, 6 in Experiment 1, 2 in Experiment 2 and 4 in Experiment 3 were excluded from the analyses based on performance near chance level. All participants reported normal or corrected-to-normal vision and confirmed a written consent before participation.

### Stimulus display

The stimuli were black figures (5.5 cd/m^2^; RGB colour code: 0, 0, 0; luminance values were measured with a LS-110 luminance meter (Minolta, Osaka, Japan) and stem from an exemplary laptop display, however, due to the nature of the online setting, variations between participants are to be expected) on a grey background (55,7 cd/m^2^; RGB colour code: 128, 128, 128; see explanation above), consisting of a fixation dot at the centre of the computer screen, a cue made of one or two circles and a target letter displaying the number 1, 2, 3 or 4. The single cue and double cue were presented for 50 ms after a random stimulus interval of 750 – 1250 ms drawn from a uniform distribution. The target letter followed after a fixed cue-target onset asynchrony (CTOA) of 500 ms with a duration of 200 ms. The stimulus sizes were adjusted to the monitor proportions to accommodate that participants performed the experiments on their monitors. Given a standard 15.6-inch display at the instructed viewing distance of 65 cm, the diameters of the black figures approximate to 0.18° of visual angle for the fixation dot and 0.62° of visual angle for the cues presented at a distance of approximately 3.8° of visual angle from centre screen.

### Procedure

**Figure 1:**
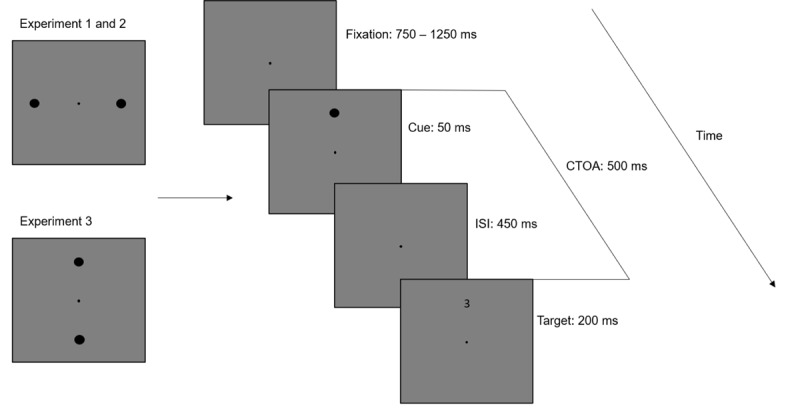
Trial sequence. Participants fixated the centre of the screen and either no cue, a single cue or a double cue was presented, after which the target appeared. In Experiments 1 and 2, the double cue appeared adjacent to the fixation point. In Experiment 3, the double cue appeared above and below the fixation point. Participants responded by pressing the mouse button corresponding to the classification of the target letter (even, odd).

Figure 1 illustrates the trial sequence for all three experiments. Participants performed a digit classification to either even or odd as quickly as possible with an external computer mouse placed in front of them. In Experiment 1, the trials were split into three cue conditions (no cue, single cue, double cue). In the alerting condition (32% of trials), two circles appeared adjacent to the fixation point prior to target onset. In the orienting condition (32% of trials), one circle appeared orthogonally to the alerting condition at one of the two target locations above or below the fixation point. In the no cue condition (32% of trials), no cues were presented. In 4% of trials no target (i.e., catch trials) was presented to reduce the number of anticipatory responses. Participants were randomly assigned to one of two block orders, mirrored at the middle of the experiment (ABBA, BAAB) to cancel out fatigue or training effects in block comparisons (cf. Poth et al., 2014). Each block provided enough time to adapt to the stimulus informativeness provided by the single cues. Block A contained the predictive cues with 100% valid trials and block B contained the anti-predictive cues with 0% valid trials. Each block started with a training of 8 practice trials followed by 150 experimental trials at a time. Between blocks participants were given short breaks. In total each experiment consisted of 632 trials lasting around 30 minutes. In Experiment 2, the design and procedure were identical to Experiment 1 except for the block manipulation. Block A contained the informative single cues with 80% valid trials, and block B contained the uninformative single cues with 50% valid trials. Experiment 3 is a replication of Experiment 2, except for changes in the alerting condition. The double cue was presented above and below the fixation point at the same position as the single cue (see Figure 1).

#### Sample size

Prior to data collection, we computed a power analysis based on the results of Lin and Lu. However, for Experiment 2, our sample size did not reach the one that had been planned in advance. Due to practical constraints at the time of testing, we decided to terminate data collection for our within-subjects design at a sample size twice the one for Lin and Lu’s between-subjects design (2016; while we matched sample sizes across Experiment 1 and Experiment 2). Therefore, we run another power analysis using the R-package pwr (1.3-0; Champely et al., 2018) based on the mean differences of the orienting effect between the informativeness blocks in Experiment 2 to determine the sample size for Experiment 3. Given a power of 0.95 and a Cohen’s *d_z_* effect size (Cohen, 1988) of 0.408 resulted in a minimum sample size of 66 participants. We planned and preregistered a sample size of 70 participants (rounding up from the 66 participants to allow for potential exclusions etc.).

### Preregistration

Experiments 2 and 3 were preregistered before data collection on the Open Science framework (https://osf.io/r4k3m). The preregistration protocol describes the initial hypotheses, study design, data collection procedures, experimental variables, sample size, data exclusion criteria, and statistical analyses.

## Results and discussion

All data sets and analysis scripts are available on the Open Science Framework (https://osf.io/xjfgq/). Statistical analyses were performed in R (4.0.5, R Core Team, 2021). Reaction times and accuracy rates for all experimental conditions were compared with repeated-measures analyses of variance with type-III sums of squares using the R-package ez (4.4.0; Lawrence, 2016) and \eta _G^2 as effect size (Bakeman, 2005). When sphericity was violated, the Huynh-Feldt correction was applied. The main effects and interaction effects were followed up by paired *t*-tests with Cohen’s *d_z_* effect size (Cohen, 1988). For the *t*-tests following up on specific main effects, we collapsed participants’ data in the conditions of the orthogonal factor (e.g., to compare reaction times in the single cue and no cue conditions, participants’ data in the two predictiveness blocks (in Experiment 1) were collapsed before their mean reaction times in the single and no cue conditions were computed, after which these mean reaction times were then subjected to the *t*-test). As a sanity check for the block manipulation, we performed paired *t*-tests, assuming greater orienting effects (Experiment 1: double – single; Experiments 2 and 3: invalid – valid) for informative than for uninformative blocks. Note that the orienting effect in Experiment 1 has been computed with the alerting cue condition as baseline because there are no validity effects with 100% and 0% trials. Complementary to the null hypothesis significance testing, JZS Bayes factors with *r* = 1 (Rouder et al., 2009) using the R-package BayesFactor (0.9.12–4.2; Morey & Rouder, 2021) have been computed for the alerting effect differences between blocks to quantify the evidence in favour of the null hypothesis. Following recent guidelines for categorising Bayes factors (BF_01_; van Doorn et al., 2021), the evidence can be quantified as follows: < 0.3 = moderate evidence in favour of the alternative hypothesis, 0.3 to 1 = weak evidence in favour of the alternative hypothesis, 1 to 3 = weak evidence in favour of the null hypothesis, and > 3 = moderate evidence in favour of the null hypothesis. Practice trials, catch trials (4%), anticipatory responses (reaction times <= 100 ms; Experiment 1: 2%; Experiment 2: 1.4%; Experiment 3: 0.7%) and trials on which participants responded more than 2.5 SD away from the individual mean in each condition (Experiment 1: 2.7%; Experiment 2: 2.4%; Experiment 3: 2.6%) were excluded from the analyses. For the reaction time analyses, we additionally excluded trials with erroneous responses (Experiment 1: 8.1%; Experiment 2: 9.7%; Experiment: 3: 7.9%).

### Experiment 1

**Figure 2:**
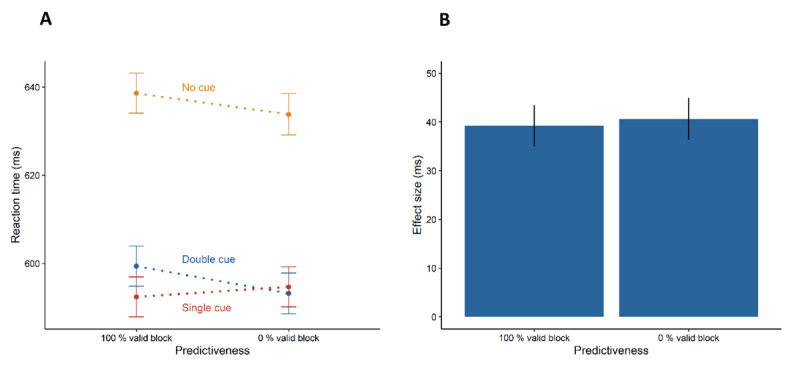
Results of Experiment 1. Left plot shows participants’ mean reaction times in the six experimental conditions. Right plot shows the alerting effect (difference between the no cue condition and the double cue condition). Error bars depict the 95% confidence intervals for within-subject designs (Morey, 2008).

Figure 2A shows the main results of Experiment 1 (see also Table 1). The repeated-measures analysis of variance with the factors cue type (no cue, single cue, double cue) and predictiveness (100% valid, 0% valid) revealed only a significant main effect of cue type, *F*(1.641, 65.640) = 103.472, *p* < .001, \eta _G^2 = 0.068. The follow-up *t*-test revealed that participants did not respond faster in trials with a preceding single cue (*M* = 593 ms, *SD* = 68 ms) than in double cue trials (*M* = 596 ms, *SD* = 75 ms), *t*(40) = –1.229, *p* = .226, *d_z_* = –0.192. The orienting effects (double – single > 0) did not differ significantly between the predictiveness blocks, *t*(40) = 1.536, *p* = .066, *d_z_* = 0.240 (although this difference was close to significance). In addition, participants responded faster in trials with a preceding single cue (see above) than in no cue trials (*M* = 636 ms, *SD* = 75 ms), *t*(40) = 10.740, *p* < .001, *d_z_* = 1.677, indicating that participants benefited from the cue. Turning to the alerting effect (Figure 2B), the analysis showed shorter reaction times in trials with a preceding double cue (see above) than in no cue trials (see above), *t*(40) = 11.423, *p* < .001, *d_z_* = 1.784. Crucially, the alerting effects did not differ between the predictiveness blocks, *t*(40) = –.250, *p* = .804, *d_z_* = -0.039. The BF_01_ = 7.964 indicated that the effects were equally strong. Thus, this finding shows that alerting was not influenced by the predictiveness of whether single cues in the current block were 100% valid or 0% valid.

**Table 1:** Mean of participants’ mean reaction time (RT) and mean of participants’ proportion of correct responses (ACC) for each experimental condition.


EXPERIMENT	RT (ms) Mean (SD)				ACC (%) Mean (SD)			

Experiment 1	No cue	Double cue	Single cue		No cue	Double cue	Single cue	

100%	640 (76)	600 (77)	592 (69)		94.0 (3.7)	91.9 (4.3)	92.4 (4.6)	

0%	632 (75)	591 (76)	592 (73)		91.8 (8.6)	90.6 (8.7)	90.6 (8.7)	

Experiment 2	No cue	Double cue	Invalid cue	Valid cue	No cue	Double cue	Invalid cue	Valid cue

80%	617 (80)	583 (82)	613 (88)	569 (80)	90.4 (8.7)	89.4 (8.6)	89.3 (10.2)	90.1 (9.2)

50%	617 (78)	583 (77)	601 (83)	577 (73)	91.6 (8.1)	89.8 (8.8)	90.0 (8.3)	90.6 (8.2)

Experiment 3	No cue	Double cue	Invalid cue	Valid cue	No cue	Double cue	Invalid cue	Valid cue

80%	610 (67)	576 (70)	595 (76)	562 (65)	92.5 (5.0)	91.4 (6.2)	91.2 (8.9)	92.2 (6.0)

50%	617 (69)	583 (72)	594 (75)	567 (68)	93.1 (4.9)	91.5 (5.5)	91.8 (6.2)	91.9 (5.5)


*Note*: Standard deviations of the means appear in parentheses.

Complementary to the reaction time analyses, we examined participants’ accuracy in our experimental conditions. The repeated-measures analysis of variance revealed a significant main effect of cue type, *F*(2, 80) = 7.940, *p* < .001, \eta _G^2 = 0.012. For the orienting effect, we found that participants’ accuracy in single cue trials (*M* = 91.5%, *SD* = 5.9%) was not significantly different from the accuracy in double cue trials (*M* = 91.2%, *SD* = 5.9%), *t*(40) = 0.706, *p* = .485, *d_z_* = 0.110. In contrast, participants’ accuracy was slightly lower in trials with a preceding single cue (see above) than in no cue trials (*M* = 92.9%, *SD* = 5.5%), *t*(40) = 2.875, *p* = .006, *d_z_* = 0.449, showing that a 100% valid single cue and a 0% counter-predictive single cue reduced the accuracy compared with the no cue condition. For the alerting effect, we also found slightly lower accuracy in trials with a preceding double cue (see above) than in no cue trials (see above), *t*(40) = 3.873, *p* < .001, *d_z_* = 0.605. Hence, the improved reaction times due to alerting were accompanied by a decrement in accuracy, which means that there was a speed-accuracy trade-off (Pachella, 1974; Wickelgren, 1977). However, this was not the case for the orienting effect, as the average accuracy was not lower for single cue trials than for double cue trials. Accuracy for the single cue trials was only lower as compared with the no cue conditions. This was probably the case because participants were not only cued to a specific target location but also alerted which has previously been shown to induce a speed-accuracy trade-off (McCormick et al., 2019; Posner et al., 1973). However, the speed-accuracy trade-off of the alerting effect did not differ between the predictiveness blocks, so that it cannot account for the equivalence of the alerting effects on reaction time.

The results of Experiment 1 show that alerting was not affected by the predictiveness blocks. Double cues equally triggered phasic alertness in 100% valid and 0% valid trials. In both predictiveness blocks the mean alerting effect was about 40 ms. If indeed alerting is mediated by current attentional priorities, participants should have had greater benefits in double cue trials with 100% validity than with 0% validity. Critically, however, trials with 0% validity always indicated the location opposite to where the target was going to appear. For this reason, participants always knew where to shift their attention. In sum, these findings argue that alerting is independent from the attentional set for orienting as manipulated by stimulus predictiveness (predictive vs. counter-predictive), but they cannot speak to effects of stimulus informativeness. Therefore, Experiment 2 investigated if phasic alerting was differentially affected by attentional sets for orienting in blocks with informative (80% validity) vs. uninformative (50% validity) single cues.

### Experiment 2

**Figure 3:**
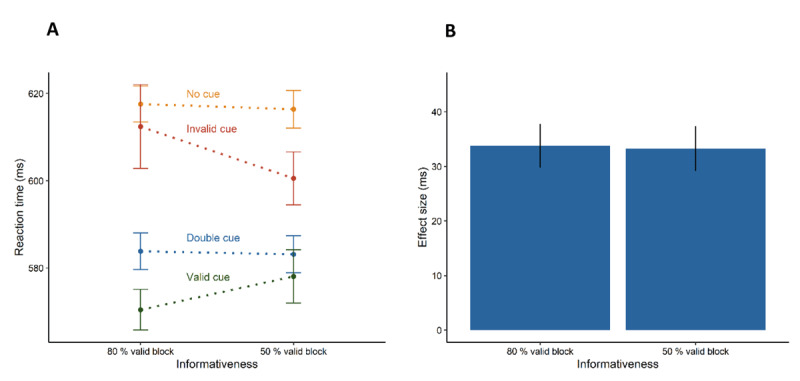
Results of Experiment 2. Left plot shows participants’ mean reaction times in the eight experimental conditions. Right plot shows the alerting effect (difference between the no cue condition and the double cue condition). Error bars depict the 95% confidence intervals for within-subject designs (Morey, 2008).

The results of Experiment 2 are visualised in Figure 3A (see also Table 1). We ran the same analyses as in Experiment 1. The repeated-measures analysis revealed a significant main effect of cue type (no cue, invalid cue, valid cue, double cue), *F*(2.004, 82.167) = 63.274, *p* < .001, \eta _G^2 = 0.046, and a significant interaction between cue type and informativeness (80% valid, 50% valid), *F*(2.202, 90.270) = 4.389, *p* = .013, \eta _G^2 = 0.002. Here, we found the classic orienting effect: shorter reaction times for trials with a preceding valid cue (*M* = 572 ms, *SD* = 76 ms) than for trials with a preceding invalid cue (*M* = 604 ms, *SD* = 83 ms), *t*(41) = 7.504, *p* < .001, *d_z_* = 1.158. In contrast to Experiment 1, the follow-up *t*-test showed that the orienting effect (invalid – valid > 0) differed between blocks, *t*(41) = 2.643, *p* = .006, *d_z_* = 0.408. Thus, the informativeness block manipulation indeed modulated the attentional set for orienting. The mean difference in reaction times between invalid and valid trials was greater in the 80% valid block compared with the 50% valid block. In addition, we found shorter reaction times in trials with a preceding valid cue (see above) than in no cue trials (*M* = 617 ms, *SD* = 78 ms), *t*(41) = 15.988, *p* < .001, *d_z_* = 2.467, and shorter reaction times in trials with a preceding invalid cue (see above) than in no cue trials (see above), *t*(41) = 3.127, *p* = .003, *d_z_* = 0.482, showing that participants benefited from preceding valid cues as well as invalid cues. The analysis also revealed shorter reaction times in trials with a preceding double cue (*M* = 583 ms, *SD* = 79 ms) than in no cue trials (see above), *t*(41) = 13.809, *p* < .001, *d_z_* = 2.131, demonstrating the classic alerting effect. Across the informativeness conditions, the alerting effects did not differ significantly between 80% valid and 50% valid trials, *t*(41) = .101, *p* = .920, *d_z_* = 0.016, whereas the BF_01_ = 8.266 in fact indicated the equivalence of the alerting effects.

Turning to the accuracy, the repeated-measures analysis of variance revealed a significant main effect of cue type *F*(3, 123) = 2.865, *p* = .039, \eta _G^2 = 0.005. For the orienting effect, we found that participants’ accuracy did not differ between trials with valid cues (*M* = 90.3%, *SD* = 7.6%) and invalid cues (*M* = 89.8%, *SD* = 7.7%), *t*(41) = –0.786, *p* = .436, *d_z_* = –0.121. Similarly, participants’ accuracy did not differ between trials with valid cues (see above) and no cue trials (*M* = 91.0%, *SD* = 7.0%), *t*(41) = 1.408, *p* = .167, *d_z_* = 0.217. In contrast, the pairwise comparisons revealed a slightly lower accuracy in trials with a preceding invalid cue (see above) than in no cue trials (see above), *t*(41) = 2.463, *p* = .018, *d_z_* = 0.380, and slightly lower accuracy in trials with a preceding double cue (*M* = 89.6%, *SD* = 7.6%) than in no cue trials (see above), *t*(41) = 3.261, *p* = .002, *d_z_* = 0.503. Therefore, we also found a speed-accuracy trade-off of the alerting effect but not the orienting effect. Crucially, the speed-accuracy trade-off did not differ between the informativeness blocks.

As in Experiment 1, single cues and double cues led to faster responses. Even though we found that orienting was modulated by the attentional set, we did not find an alerting interaction. In both informativeness blocks, the mean alerting effect was about 33 ms. Thus, taken together, these findings indicate that in contrast to orienting, phasic alerting does not seem to be influenced by the attentional set for orienting. Experiment 2 focused on non-spatial features by separating alerting and orienting cues on different visual axes, in contrast to the previous study that found an alerting-informativeness interaction (Lin & Lu, 2016). Thus, one might suppose that alerting and the informativeness of the cue stimuli for orienting only interact when both cue types are presented at the same visual locations. Therefore, we conducted Experiment 3 which used the same informativeness manipulation as Experiment 2 but presented all cues at the same locations.

### Experiment 3

**Figure 4:**
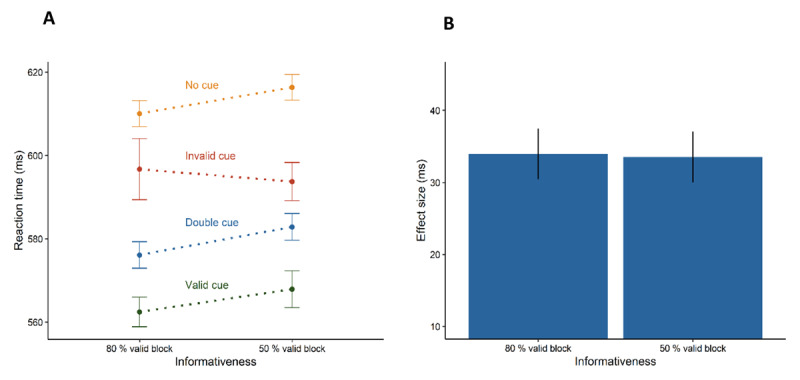
Results of Experiment 3. Left plot shows participants’ mean reaction times in the eight experimental conditions. Right plot shows the alerting effect (difference between the no cue condition and the double cue condition). Error bars depict the 95% confidence intervals for within-subject designs (Morey, 2008).

The results of Experiment 3 are visualised in Figure 4A (see also Table 1). The analyses were identical to Experiment 1 and Experiment 2. The repeated-measures analysis only revealed a significant main effect of cue type, *F*(2.591, 181.400) = 101.830, *p* < .001, \eta _G^2 = 0.063. Reaction times were shorter in trials with a preceding valid cue (*M* = 564 ms, *SD* = 65 ms) than with a preceding invalid cue (*M* = 594 ms, *SD* = 74 ms), *t*(70) = 9.681, *p* < .001, *d_z_* = 1.149, demonstrating the orienting effect. As in Experiment 2, the follow-up *t*-test showed that the orienting effect (invalid – valid > 0) differed between the informativeness blocks, *t*(70) = 1.674, *p* = .049, *d_z_* = 0.199, so that the mean difference in reaction times between invalid and valid trials was greater in the 80% valid block compared with the 50% valid block. Again, similar to Experiment 2, reaction times were also shorter in trials with a preceding valid cue (see above) than in no cue trials (*M* = 614 ms, *SD* = 67 ms), *t*(70) = 16.884, *p* < .001, *d_z_* = 2.004, as well as shorter in trials with a preceding invalid cue (see above) than in no cue trials (see above), *t*(70) = 6.186, *p* < .001, *d_z_* = 0.734. The analysis also revealed shorter reaction times in trials with a preceding double cue (*M* = 579 ms, *SD* = 70 ms) than in no cue trials (see above), *t*(70) = 12.754, *p* < .001, *d_z_* = 1.514, demonstrating the alerting effect. There were no differences between the alerting effects for the informativeness blocks with 80% validity and 50% validity of the orienting cues, *t*(70) = 0.130, *p* = .897, *d_z_* = 0.015, and again, the BF_01_ = 10.616 suggested the equivalence of the alerting effects in the informativeness blocks.

The repeated-measures analysis of variance for the response accuracy revealed a significant main effect of cue type *F*(2.107, 147.502) = 3.976, *p* = .019, \eta _G^2 = 0.008. On average participants’ accuracy was not significantly different between trials with valid orienting cues (*M* = 92.1%, *SD* = 5.1%) and invalid orienting cues (*M* = 91.6%, *SD* = 5.9%), *t*(70) = –0.947, *p* = .347, *d_z_* = –0.112. In contrast, accuracy was lower in trials with a preceding valid cue (see above) than in no cue trials (*M* = 92.8%, *SD* = 4.3%), *t*(70) = 2.245, *p* = .028, *d_z_* = 0.266. The pairwise comparisons also revealed lower accuracy in trials with a preceding invalid cue than in no cue trials (see above), *t*(70) = 2.370, *p* = .021, *d_z_* = 0.281, and with a preceding double cue (*M* = 91.4%, *SD* = 5.1%) than in no cue trials (see above), *t*(70) = 4.311, *p* < .001, *d_z_* = 0.512. As in Experiments 1 and 2, we found a speed-accuracy trade-off only for the alerting effect, which however did not differ between the informativeness blocks.

In summary, responses were also sped up with single cues and double cues. Although the difference in the orienting effects between the informativeness blocks was smaller in Experiment 3 than in Experiment 2, we found that orienting was modulated by the attentional set. As in the previous experiments, however, we observed about the same alerting effects in both informativeness blocks, namely alerting effects of about 34 ms.

## General Discussion

The present study investigated how the current attentional set for orienting affects phasic alerting. In three experiments, we found that alerting was not modulated by the attentional set for orienting, as manipulated by changing the predictiveness and informativeness for orienting associated with stimuli used as alerting cues. Replicating classic findings (Eriksen & Yeh, 1985; Johnson & Yantis, 1995; Riggio & Kirsner, 1997), performance was better when targets were preceded by orienting cues with high informativeness. With uninformative cues, the beneficial effects of the orienting cues were much smaller. In contrast to these observed effects, alerting cues facilitated performance across all predictiveness or informativeness conditions about equally. Experiment 1 revealed that the alerting effect was unaffected when the stimuli used as alerting cues were associated with informative orienting cues with validities of 0% (informative but counter-predictive) compared with 100% (informative and predictive). Experiment 2 showed the same pattern also for alerting stimuli associated with completely uninformative cues with 50% validity compared with informative cues with 80% validity. Experiment 3 replicated the findings of Experiment 2 by using the same spatial locations for both cue types. Taken together, the present findings argue that phasic alertness is either completely unaffected by the attentional set for spatial orienting or affected to a much lesser degree than orienting.

In Experiment 1, the attentional set was manipulated by means of completely predictive and counter-predictive single cues. It has been shown that when the target location is always known, spatial attention is highly focused towards that location (Folk et al., 1992). Thus, the completely predictive single cues should have determined the attentional set for orienting and, as a consequence, the allocation of spatial attention. However, alerting effects were comparable for double cues whose stimuli were associated with predictive or counter-predictive single cues. This suggests that phasic alertness was independent from the attentional set for orienting, whose representations of orienting cues matched the alerting cues in terms of their non-spatial surface features. Both the predictive and counter-predictive single cues informed about the location of the upcoming target. Predictive cues indicated the target location directly, counter-predictive cues indicated the target location by exclusion of the cued location. Thus, both single cues were predictive of the target location, and this might be the reason why there were no differential effects on orienting, and in turn alerting. This is in line with a previous study that found comparable orienting effects under these conditions (Eckstein et al., 2004). Hence, it provides a hint that it is the informativeness and not the spatially specific prediction of a target that is used within the attentional set for orienting. Therefore, Experiment 2 was conducted with blocks in which orienting cues were valid on 80% or 50% of the trials. This experiment also yielded equal alerting effects, even though stimuli of double cues were associated with informative compared with uninformative single cues. As outlined before, we presented double cues and single cues on different visual axes, so that both cue types appeared at different locations. This was in contrast to a previous study that found alerting to be modulated by the informativeness of orienting cues (Lin & Lu, 2016). Thus, one might suspect effects of the attentional set for orienting on alerting, but only for situations in which orienting cues and alerting cues share common spatial locations. Therefore, Experiment 3 presented double and single cues at the same locations. Again, it was found that the alerting effects were about equal across the informative and uninformative blocks. These findings conflict with those of Lin and Lu (2016), who found a clear effect showing that stimulus informativeness modulated alertness. This was the case even though they used a between-subjects design, which is less powerful for detecting interactions between the informativeness for orienting and alerting than our within-subjects design (Charness et al., 2012). One should note, however, that our within-subjects design may have led to carry-over effects of the respective predictiveness and informativeness conditions which were eliminated by the between-subjects design of Lin and Lu (2016). Thus, it is possible that our participants were still expecting a certain validity of orienting cues after a block of trials because it took more exposures to the current validity of orienting cues to adapt the attentional set for orienting. This might have caused smaller effects of the attentional set for orienting compared with Lin and Lu’s study, because attentional sets for the current validity of the orienting cues could have still been in the process of building up. Critically, however, such potential carry-over effects of previous blocks should have been minimised in our design, because participants performed a number of practice trials before each new informativeness block that should have allowed them to learn the new validity of the orienting cues. Moreover, we randomised the two block orders between participants and mirrored them at the middle of the experimental session to cancel out training or fatigue effects (cf. Poth et al., 2014), which should also control for effects of block order.

To date, evidence for top-down influences on phasic alertness is only alluded through temporal expectancy (Weinbach & Henik, 2012). In contrast, spatial orienting effects are clearly affected by current goals and task-derived processing priorities (Corbetta et al., 2000; Desimone & Duncan, 1995; Kastner & Ungerleider, 2000). Interestingly, Lin and Lu’s findings suggest phasic alertness is to some extent determined by top-down expectations of spatial orienting (Lin & Lu, 2016). Indeed, salient visual stimuli near the target location with short CTOAs have been shown to produce effects of orienting and alerting at the same time (Chica et al., 2011). But, it has been argued that such hybrid cues trigger both, bottom-up and top-down processes independently (Botta et al., 2014). Similarly, Lin and Lu proposed that phasic alertness is influenced by a top-down process shared with temporal expectation (Lin & Lu, 2016). Salient visual stimuli inherently contain some temporal information, which may allow for top-down temporal preparation (Weinbach & Henik, 2012). However, it has been shown that alerting cues boost performance even if the temporal expectation was kept constant by drawing CTOAs from non-aging probability distributions (Petersen et al., 2017; Weinbach & Henik, 2013). Thus, even though both may occur simultaneously, phasic alertness does not seem to depend on temporal expectation (Weinbach & Henik, 2012). Following established paradigms of phasic alerting (Callejas et al., 2005; Fan et al., 2002; Ishigami & Klein, 2010), the present experiments used a constant CTOA. This allowed for fewer trials and shorter experiments, but as a consequence, temporal expectation should have contributed to our alerting effects (Nobre & van Ede, 2017). It is important to note, however, that this does not invalidate the present findings. That is, even if the findings reflected an alerting process as well as temporal expectation processes, they still argue against their modulation by the attentional set for orienting.

For our constant CTOA of 500 ms, one could have assumed that valid orienting decreased performance. That is because for such a CTOA, inhibition of return can occur, which is the impairment of performance for targets at cued locations (and the facilitation of performance for targets at uncued locations) due to the disengagement of attention after a short period of time (Klein, 2000). However, we did not observe inhibition of return, since our valid orienting cues always yielded shorter reaction times than invalidly cued trials, indicating that the cues still drew attention to the cued location. In discrimination tasks, inhibition of return effects are assumed to occur later as compared with the easier detection tasks (Chica et al., 2014; Klein, 2000; Lupiáñez et al., 1997). This is in line with our reaction time findings showing that participants indeed benefited from the valid cues with our particular CTOA. The accuracy results also argue for an engagement of the orienting mechanism. Similar to previous studies on orienting (Callejas et al., 2005; Fan et al., 2002), we did not find a speed-accuracy trade-off accompanying the orienting effect. However, we did find speed-accuracy trade-offs when we compared the conditions with cues to the no cue conditions. That is, in the cue conditions, faster responses were accompanied by a lower response accuracy. In all of these conditions, targets were preceded by the cues, so that the cues could always function as alerts for the targets. Thus, even orienting cues drawing spatial attention to or away from the targets should have included an alerting component. Therefore, since alerting has been shown to induce a speed-accuracy trade-off (McCormick et al., 2019; Posner et al., 1973), we believe that the speed-accuracy trade-offs we observed were in fact due to the alerting component in our conditions with cues. It is important to note, however, that this does not explain our main findings, namely that alerting was unaffected by the predictiveness or informativeness of the orienting cues. Specifically, if a speed-accuracy trade-off caused these results, then the reaction time benefits due to alerting should have been accompanied by different decrements in accuracy in the two predictiveness blocks or informativeness blocks, which was not the case. For studying orienting, however, the lower accuracy in the conditions with cues highlights that one should keep alerting constant when examining orienting because the speed-accuracy trade-off induced by alerting could otherwise lead to false interpretations.

The present data also converge with neurophysiological and neuropsychological studies supporting the idea of separate attentional networks for alerting and orienting mechanisms (Fan et al., 2005; Petersen & Posner, 2012; Posner & Petersen, 1990; Raz & Buhle, 2006; Thiel et al., 2004). The orienting network is mainly located in frontal and posterior parts of the human brain (Petersen & Posner, 2012). In particular, the frontal eye fields seem to play a major role in the selection and prioritisation of relevant stimuli (Corbetta et al., 1998). The alerting network is thought to be implemented in frontal and parietal areas (Petersen & Posner, 2012). Accumulating evidence suggests that alerting is driven by the locus coeruleus-norepinephrine system which is responsible for maintaining an adequate level of arousal (Aston-Jones & Cohen, 2005). The activity of the locus coeruleus-norepinephrine system has been associated with different modes (tonic and phasic; Aston-Jones & Cohen, 2005; Gabay et al., 2011; Gabay & Henik, 2010) as reflected by pupillary responses through state changes of phasic alertness (Petersen et al., 2017) and urgency (Poth, 2021). This in turn has been found to influence the appearance of inhibition of return, a common phenomenon of spatial orienting (Gabay et al., 2011), which demonstrates that both networks can interact. At the behavioural level, it has also been shown that these structures can work in concert (i.e., additive effects or interaction effects) but seem to be independent from another (Callejas et al., 2005; Fan et al., 2002; Fernandez-Duque & Posner, 1997). These previous findings agree with the present data that the alerting network is separate as it does not depend on the attentional set controlling orienting.

## Conclusion

The present findings provide a simple dissociation of phasic alertness from the attentional set used for orienting spatially selective attention. Even though attentional sets based on stimulus predictiveness and informativeness affected orienting, they did not affect the effectiveness of stimuli as alerting cues. In this way, the present findings reveal that the mechanisms exerting top-down control on spatial attention leave the mechanisms for phasic alerting untouched.

## Data Accessibility Statements

The data of the experiments, the R-scripts for the analyses and the figures provided in the manuscript have been uploaded to the Open Science Framework: https://osf.io/xjfgq/.
